# Nuclear Accumulation of Bm65 Aggregate Is Blocked by Mutations in the Nuclear Export Sequence of Bm65

**DOI:** 10.3390/v17020248

**Published:** 2025-02-12

**Authors:** Guohui Li, Wenchao Liu, Yunyun Liu, Junting Xu, Huiqing Chen, Feifei Zhu, Zhaoyang Hu, Zhongjian Guo, Keping Chen, Qi Tang

**Affiliations:** School of Life Sciences, Jiangsu University, 301# Xuefu Road, Zhenjiang 212013, China; ghli@ujs.edu.cn (G.L.); 15726383271@163.com (W.L.); yunyliu88@163.com (Y.L.); juntingxu2023@163.com (J.X.); sw98318@21cn.com (H.C.); feifzhu@ujs.edu.cn (F.Z.); sunnyhu163@163.com (Z.H.); gzh762677@ujs.edu.cn (Z.G.); kpchen@ujs.edu.cn (K.C.)

**Keywords:** *Bm*NPV Bm65, tetramer, nuclear export signal, Bm65 truncation

## Abstract

A nuclear export signal (NES) is a cluster of hydrophobic amino acids that can maintain the dynamic shuttling of target proteins between the nucleus and cytoplasm. Bioinformatics analysis showed that the hydrophobic region of ^92^PLLLHKFLLA in Bm65 is very likely to be an NES and may be involved in the production of infectious virions. In this study, we generated several mutations in ^92^PLLLHKFLLA of Bm65, which were further used to generate recombinant viruses to study their roles in viral propagation. Subcellular analysis revealed that the ^92^PLLLHKFLLA sequence was an NES involved in the dynamic transport of Bm65. Mutations in the hydrophobic region could block the formation and accumulation of Bm65 aggregates, resulting in a uniform distribution of Bm65 in BmN cells. The ribosomal protein L13 (RPL13) of silkworms was previously reported to interact with Bm65. Here, intracellular co-localization analysis showed that the interaction between Bm65 and RPL13 was regulated by the ^92^PLLLHKFLLA of Bm65. In summary, the interaction between Bm65 and RPL13 is essential for the production and accumulation of Bm65 aggregates and may play an important role in the regulation of viral propagation.

## 1. Introduction

*Bombyx mori* nucleopolyhedrovirus (BmNPV) *orf65* (Bm65) is expressed at the early stages of BmNPV infection in BmN cells and is involved in the production of progeny virions. Previous studies have shown that Bm65 plays important roles in DNA damage repair, the high efficiency of viral replication, and the high production of progeny virions during the viral life cycle [1,2,3]. *Bm65* codes for a protein comprising 104 deduced amino acid residues with a molecular mass of approximately 12.2 kDa. Therefore, it is regarded as a relatively small protein (<40 kDa) that can, in theory, passively diffuse between the cytoplasm and nucleus [4,5]. Surprisingly, Bm65 was reported to be located mainly in the nucleus, with a small proportion in the cytoplasm, and Bm65 was clustered into visible aggregates with some unknown components in the nucleus [1]. Moreover, Li et al. reported the ^76^KRKCSK sequence to be an efficient nuclear localization signal of Bm65 that was directly involved in viral production [6]. To date, the mechanism by which Bm65 aggregates in the nucleus and the dynamic shuttling of Bm65 between the nucleus and cytoplasm remain unclear. Therefore, it is necessary to further study the correlation of the Bm65-RPL13 interaction and the formation of Bm65 aggregates.

Previous studies have shown that Bm65 can form aggregates and accumulate in the nuclei of BmNPV-infected BmN cells [1,3]. Similar studies have reported that some proteins aggregate in the nucleus or cytoplasm of virus-infected cells [7,8,9,10]. Various cellular factors can affect protein aggregate accumulation and regulate viral propagation and cellular function, even in the occurrence of diseases in humans [11,12]. However, the mechanism by which Bm65 accumulates in the nuclei of BmNPV-infected cells is unclear. It is speculated that host proteins are indispensable for the formation of Bm65 aggregates in the nucleus. Therefore, it is necessary to identify scientific issues concerning the formation mechanism of Bm65 aggregates and the correlation between Bm65 aggregates and viral propagation.

A leucine-rich region was clustered at the C-terminus of Bm65, which was predicted to be a potential nuclear export signal (NES) using NetNES1.1 software. To date, the function of the leucine-rich region of Bm65 is unclear. NESs play an important role in maintaining the dynamic shutting balance of target proteins between the cytoplasm and nucleus and are also involved in the functional regulation of target proteins. Leucine-rich NESs are among the most widely studied signals that can be recognized by chromosome region maintenance 1. Human immunodeficiency virus (HIV) Rev was the first identified NES-containing protein [13,14]. To date, all identified NESs are leucine-rich, and they are often involved in intracellular biochemical processes, such as gene expression, signal transduction, autophagy, viral replication, and infection [15,16,17,18]. However, incorrect protein localization can cause cellular abnormalities involving protein aggregation, biosynthesis, and metabolic disorders, resulting in the occurrence of some diseases in humans [19,20].

The putative NES was amplified from the Bm65 sequence and fused with enhanced green fluorescent protein (EGFP), which was used to study the intracellular distribution of Bm65. Further research was conducted to study the effect of Bm65 NES on viral propagation. Additionally, the ribosomal protein L13 (RPL13), in conjunction with rRNA, was reported to interact with Bm65 via Co-Immunoprecipitation and Liquid Chromatograph–Mass Spectrometer (LC-MS/MS) analysis [21]. RPL13 is a major component of ribosomes involved in protein biosynthesis. Thus, Bm65 NES mutations were created to demonstrate the binding site of Bm65 with RPL13. Meanwhile, the mutations were also used to further study the role of Bm65 NES in the viral life cycle. In a word, some mutations in the Bm65 NES were made to study the effect on the production and accumulation of Bm65 aggregates and viral propagation.

## 2. Materials and Methods

### 2.1. Recombinant Viruses, Plasmids, and Cells

A BmNPV bacmid (Bm-bacmid) with a deletion of Bm65 (Bm^Bm65KO^) was constructed using homologous recombination and maintained in our laboratory [3]. Two Bm-Bacmids of the wild type and *Bm65*-deleted type harboring the pMON7124 helper plasmid were propagated in the *Escherichia coli* strain DH10B, which can be used to produce recombinant viruses through transposition. Three recombinant plasmids, namely pMD18T-P_Bm65_-Bm65-flag, HTB-P_ie1_-EGFP, and HTB-P_ie1_-EGFP-sv40-P_Bm65_-Bm65-flag, were maintained in our laboratory [3]. Some viruses, including wild-type BmNPV, vBm^Bm65(NES-M1)^, vBm^Bm65(NES-M2)^, vBm^Bm65(NES-M3)^, vBm^Bm65M10^, and vBm^Bm65M11^, were generated for further research in the study. Plasmids of HTB-P_ie1_-Bm65NES-EGFP and HTB-P_ie1_-Bm65(NES-M3)-EGFP were constructed to study the intracellular distribution of fluorescence signals. BmN cells were used for transfection or infection in the study.

### 2.2. Computer-Assisted Sequence Analysis

The NetNES1.1 software (http://www.cbs.dtu.dk/services/NetNES/)(accessed on 20 December 2022) was used to predict NESs in Bm65 sequences. The PSIPRED server (http://bioinf.cs.ucl.ac.uk/psipred/) (accessed on 24 December 2022) was used to predict the secondary structure of Bm65.

### 2.3. Transfection and Fluorescence Microscopy

BmN cells (10^6^ cells/dish) were seeded into 35 mm dishes and incubated at 27 °C for 16–24 h before transfection. Recombinant DNA molecules (2 µg/dish) and 5 µL Cellfectin (Invitrogen Life Technology, Carlsbad, CA, USA) were mixed in a total volume of 200 µL TC-100 serum-free medium, followed by an additional 45 min of incubation at 27 °C. Then, 800 µL serum-free medium was added to the DNA–Cellfectin solution, before being finally overlaid onto BmN cells, followed by 5 h of additional incubation at 27 °C. After the incubated cells were washed with serum-free TC-100 medium, 2 mL of TC-100 medium containing 10% fetal bovine serum was added to each dish for further culture. Fluorescence microscopy (Olympus-IX73-DP80, Tokyo, Japan) was used to observe fluorescence signals in BmN cells expressing target proteins at selected time points.

### 2.4. Recombinant Viruses for Expression of Bm65 and Bm65 Mutants

Several mutations, including ^92^P(E)L(E)L(E)L(E), ^98^F(E)L(E)L(E)L(E), and ^92^P(E)L(E)L(E)L(E)^98^F(E)L(E)L(E)L(E), were introduced in the Bm65 sequence according to the instructions of the MutanBEST Kit (TaKaRa, Tokyo, Japan). Briefly, pMD18T-P_Bm65_-Bm65-flag was used as a template to introduce ^92^P(E)L(E)L(E)L(E) and ^98^F(E)L(E)L(E)L(E) in the Bm65 sequence using primer pairs of Bm65-flag-F/NES-M1-R and Bm65-flag-F/NES-M2-R, respectively. Additionally, pMD18T-P_Bm65_-Bm65 (NES-M1)-Flag, containing the mutation of ^92^P(E)L(E)L(E)L(E), was used as a template to introduce the mutation of ^92^P(E)L(E)L(E)L(E)^98^F(E)L(E)L(E)L(E) in the Bm65 sequence using Bm65-flag-F and NES-M2-R by PCR. The three recombinant plasmids were digested with *Spe*I and *Xho*I, and the resulting purified target DNA fragment was ligated with HTB-P_ie1_-egfp-sv40-P_Bm65_-Bm65-flag digested with the same enzymes to generate HTB-P_ie1_-egfp-sv40-P_Bm65_-Bm65(NES-M1/M2/M3)-Flag. Transposition between HTB-P_ie1_-egfp-sv40-P_Bm65_-Bm65(NES-M1/M2/M3)-Flag and Bm^Bm65KO^ was introduced to generate Bm^Bm65(NES-M1/M2/M3)-Flag−GFP^, which was selected by blue–white screening and further confirmed by PCR using M13 primers. pMD18T-P_Bm65_-Bm65(NES-M3)-Flag was used as a template to introduce ^33^R(A)R(A)I(A)K(A) into the Bm65(NES-M3) sequence using Bm65(M11)-F and Bm65(M11)-R. Subsequently, HTB-P_ie1_-egfp-sv40-P_Bm65_-Bm65(M10/M11)-Flag was constructed via enzyme digestion and ligation. Additionally, Bm^Bm65M10^ and Bm^Bm65M11^ were generated by transposition between Bm^Bm65KO^ and HTB-P_ie1_-egfp-sv40-P_Bm65_-Bm65(M10/M11)-Flag using blue–white screening and PCR confirmation.

### 2.5. Construction of Recombinant Plasmids

Bm65NES-F and Bm65NES-R were used to amplify truncated Bm65 fused with EGFP from HTB-P_ie1_-Bm65-EGFP to produce Bm65NES-EGFP. The DNA fragment was ligated with HTB-P_ie1_-Bm65-EGFP digested with *EcoR*I and *Xho*I to produce HTB-P_ie1_-Bm65NES-EGFP. pMD18T-P_Bm65_-Bm65(NES-M3)-Flag was used as a template to amplify a 79 bp Bm65NES(M3) fragment using Bm65NES-F and Bm65NES(M3)-R, which was ligated with HTB-P_ie1_-Bm65-EGFP and digested with *EcoR*I and *Pst*I to generate HTB-P_ie1_-Bm65NES(M3)-EGFP.

To generate a series of Bm65 mutants in the ^33^RRIK region, pMD18T-Bm65 was used as a template to amplify Bm65 mutants with different primer pairs according to the instructions of the MutanBEST Kit (TaKaRa). Briefly, primer pairs of Bm65(M1)-F/Bm65M-R, Bm65(M2)-F/Bm65M-R, Bm65(M3)-F/Bm65M-R, and Bm65(M4)-F/Bm65M-R were used to make single point mutations in ^33^RRIK, resulting in the generation of ^33^R(A)RIK, ^33^RR(A)IK, ^33^RRI(A)K, and ^33^RRIK(A), respectively. Bm65(M5)-F/Bm65M-R, Bm65(M6)-F/Bm65M-R, and Bm65(M7)-F/Bm65M-R were used to make two point mutations in ^33^RRIK, resulting in the generation of ^33^R(A)R(A)IK, ^33^RR(A)I(A)K, and ^33^RRI(A)K(A). Bm65(M8)-F/Bm65M-R and Bm65(M9)-F/Bm65M-R were used to produce three point mutations in ^33^RRIK, resulting in the generation of ^33^R(A)R(A)I(A)K and ^33^RR(A)I(A)K(A). Bm65(M9)-F and Bm65M-R were used to introduce ^33^R(A)R(A)I(A)K(A) in the Bm65 sequence. Subsequently, these Bm65 mutants were used as a template to amplify target DNA using Bm65-F and Bm65-R, which were ligated into HTB-P_ie1_-Bm65-EGFP digested with *EcoR*I and *Pst*I to generate the corresponding recombinant plasmids.

### 2.6. Confocal Microscopy Analysis

BmN cells (1 × 10^5^) were seeded into a 35 mm glass-bottom cell culture dish (NSET), and 2 µg of each plasmid was used for transfection. Bm65 and Bm65 mutants fused with EGFP were transiently expressed in BmN cells under the control of the *ie1* promoter. The cell culture supernatants were removed 48 h after transfection (hpt), before treatment. Briefly, the cells were washed three times with phosphate-buffered saline (PBS; 2.6 mM KCl, 0.136 M NaCl, 8 mM Na_2_HPO_4_, 2 mM KH_2_PO_4_, pH 7.4), fixed with 4% paraformaldehyde for 15 min, washed three times with PBS for 10 min, and permeabilized in 0.1% Triton ×100 for 15 min. Finally, the cells were stained with DAPI (60 µg/mL, Sigma, London, UK) for 10 min and washed three times with PBS. Confocal microscopy was used to observe fluorescence signals as previously described [1,6].

### 2.7. Viral Growth Curve Analysis

Virus growth curve analysis was performed as previously described to determine the effect of Bm65 mutants on viral propagation [3]. Briefly, BmN cells (10^6^ cells/well) were seeded into six-well plates and cultured overnight. The next day, 2.0 µg of recombinant acid DNA was used for transfection of BmN cells to generate recombinant viruses. The supernatants of transfected BmN cells containing recombinant viruses were harvested at selected time points. The Budded Virus (BV) titer was determined using 50% tissue culture infective dose (TCID50) end-point dilution, as described previously [3]. The presence of green fluorescence in BmN cells indicated successful viral infection. The experiments were repeated three times. Statistical analysis was performed using a single-factor analysis of variance.

### 2.8. Quantitative Analysis of Viral DNA Synthesis

To further study the effect of mutations in ^92^PLLLHKFLLA of Bm65 on viral replication, quantitative real-time PCR (qPCR) analysis was performed to examine the copies of the viral genome in recombinant virus-infected BmN cells, as described previously [22]. Briefly, some recombinant viruses, including wild-type Bm65NES-M1, Bm65NES-M2, and Bm65NES-M3 BmNPV, were used to infect BmN cells for comparative analysis of viral genome copies. Total DNA was extracted from each sample using a Universal Genomic DNA Extraction Kit (TaKaRa) according to the manufacturer’s instructions. The total DNA was resuspended in 150 μL of sterile water. qPCR analysis was performed with 10 μL of the extracted DNA and Hot Start PCR Master Mix III (TaKaRa, Tokyo, Japan) according to the manufacturer’s instructions using qPCR-F/qPCR-R targeting the 170 bp region of *GP64*. All primers used in the study are listed in Table 1.

## 3. Results

### 3.1. A Leucine-Rich Cluster Could Mediate the Nuclear Export of Bm65

**Figure 1:**
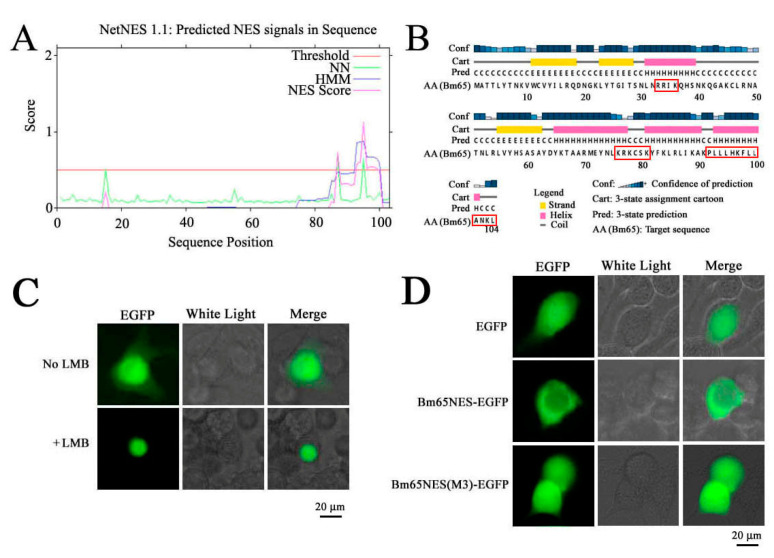
Fluorescence microscopy of BmN cells with expression of Bm65 and a truncated Bm65 mutant. (**A**) Prediction of Bm65 nuclear export signals using NetNES1.1 software. (**B**) Prediction of the secondary structure of Bm65 using PS-IPRED software. Yellow, pink, and gray symbols indicate the β-strand, α-helix, and random coils, respectively. The red box indicates the location of the predicted functional sequence in the Bm65 secondary structure. (**C**) Effect of LMB on the intracellular distribution of Bm65. (**D**) Subcellular localization of truncated Bm65 (aa: 95–104) determined via fluorescence microscopic analysis.

Hydrophobic amino acids are abundant in the C-terminal region of Bm65 (Figure 1A), and a potential NES was detected in Bm65 via analysis using NetNES1.1 software. This NES may be involved in maintaining the dynamic balance of the intracellular distribution of Bm65. Additionally, PS-IPRED software 3.5 was used to predict the secondary structure of Bm65. The results indicated that the hydrophobic amino acid cluster of ^92^PLLLHKFLLA was located in the α-helix region with high confidence (Figure 1B). However, its role in viral propagation is unclear. Thus, further studies are required to clarify the specific role of the viral life cycle.

According to the result shown in Figure 1, a possible NES was contained in the hydrophobic cluster of Bm65. To verify this hypothesis, enhanced green fluorescence protein (EGFP) fusion with Bm65 or truncated Bm65 was used to study the intracellular distribution of the target protein by observing the fluorescence signal. First, leptomycin B (LMB), an efficient nuclear export inhibitor (2 ng/mL), was used to study its inhibitory effect on the cytoplasmic transport of Bm65. The results indicated that green fluorescence signals were located mainly in the nucleus and cytoplasm. However, a green fluorescence signal was present only in the nucleus, indicating that LMB could restrain the transport of Bm65 from the nucleus to the cytoplasm (Figure 1C). Furthermore, the BmN cells treated with LMB were smaller than untreated BmN cells. Therefore, Bm65 contained a potential NES for the dynamic shuttling of Bm65 between the nucleus and cytoplasm.

To further confirm whether the hydrophobic region at the C-terminus of Bm65 possesses the function of nucleocytoplasmic transport, recombinant Bm65 protein (aa: 95–104) containing the potential NES fusion with EGFP was expressed in BmN cells. Multiple point mutations in the key amino acids of truncated Bm65 (aa: 95–104) fused with EGFP were also observed in BmN cells. The fluorescence microscopy results showed that a green fluorescence signal was present mainly in the cytoplasm of BmN cells transfected with HTB-P_ie1_-Bm65NES-EGFP, but a uniform distribution was observed in BmN cells for the expression of Bm65NES(M3)-EGFP (Figure 1D). The expression of EGFP alone in BmN cells (shown in the EGFP panel in Figure 1D) was used as the negative control. Therefore, the above results indicate that the hydrophobic region of ^92^PLLLHKFLLA is an active NES involved in nucleocytoplasmic transport.

### 3.2. Mutation of ^33^RRIK into AAAA Facilitates the Formation of Bm65 Aggregates

**Figure 2:**
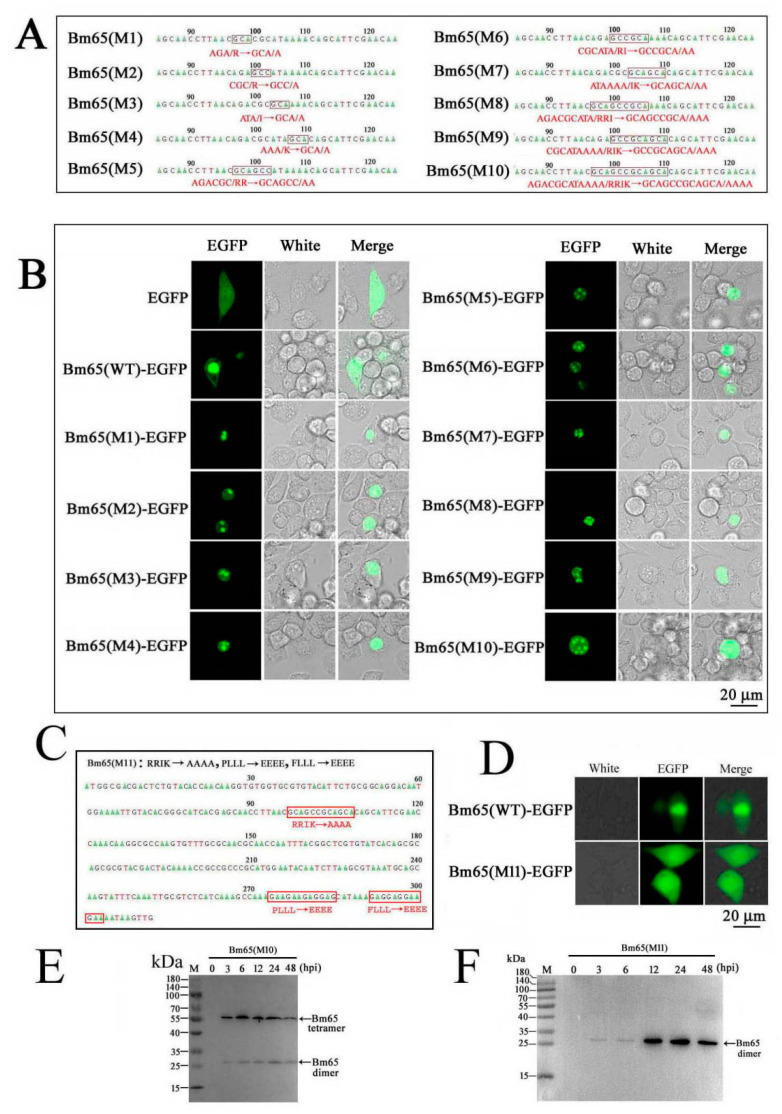
Contribution of hydrophobic amino acids to the formation of Bm65 aggregates. (**A**) Schematic diagram of Bm65 mutants with some mutations in ^33^RRIK. (**B**) Fluorescence microscopy analysis of BmN cells with expression of Bm65 and Bm65 mutants. (**C**) Sequencing result of Bm65(M11) with concurrent mutations in ^33^RRIK and ^92^PLLLHKFLLA. (**D**) Fluorescence microscopy analysis of BmN cells with expression of Bm65(M11) fusion with EGFP. (**E**) Western blot analysis of Bm65(M10) from the total protein of BmN cells infected with vBm^Bm65(M10)-Flag^. (**F**) Western blot analysis of Bm65(M11) from the total protein of BmN cells infected with vBm^Bm65(M11)-Flag^.

It was interesting to observe Bm65 aggregates in the nuclei of BmN cells infected with recombinant BmNPV in previous reports [1,2,3]. However, the formation mechanism of Bm65 aggregates remains unclear. To further study the effect of hydrophobic amino acids on the accumulation of Bm65 in the nucleus, mutations were made in the ^33^RRIK sequence of Bm65 according to the instructions of the MutanBEST Kit. The sequencing results indicated that some mutations were successfully identified in the ^33^RRIK of Bm65 (Figure 2A). Fluorescence microscopy revealed some fluorescent aggregates accumulated in the nuclei of BmN cells expressing the Bm65 mutant fusion with EGFP (Figure 2B). EGFP was used as a negative control and showed uniform distribution in BmN cells (Figure 2B). The results indicated that ^33^RRIK mutated into ^33^AAAA in Bm65 did not abrogate the formation of fluorescent aggregates but could facilitate the nuclear accumulation of Bm65 aggregates. Therefore, further research is required to demonstrate the mechanism by which Bm65 aggregates in the nucleus. In a word, none of the mutations actually affect the nuclear localization of Bm65, but the mutations make Bm65 prone to form aggregate accumulation in the nucleus.

To further study the effect of the hydrophobic region of ^92^PLLLHKFLLA on Bm65(M10) aggregates, ^92^PLLLHKFLLA was mutated into ^92^EEEEHKEEEE in the Bm65(M10) sequence according to the instructions of the MutanBEST Kit. The sequencing result indicated that a successful mutation was made in the hydrophobic region of Bm65(M10) (Figure 2C), and the mutant was termed Bm65(M11). Surprisingly, the fluorescence aggregate disappeared in BmN cells with expression of Bm65(M11)-EGFP, and the fluorescence signal was uniformly distributed in BmN cells (Figure 2D). Furthermore, two recombinant viruses of vBm^65KO-Pie1-Bm65(M10)-EGFP^ and vBm^65KO-Pie1-Bm65(M11)-EGFP^ were created, which were used to infect BmN cells to express Bm65(M10) and Bm65(M11). The Western blotting results revealed a Bm65(M10) tetramer in BmN cells infected with vBm^65KO-Pie1-Bm65(M10)-Flag^, with the presence of a weak band of a Bm65(M10) dimer from 3 to 48 hpi (Figure 2E). However, Bm65(M11) existed in a dimeric form in BmN cells infected with vBm^65KO-Pie1-Bm65(M11)-Flag^ from 12 to 48 hpi (Figure 2F). The results indicated that the mutations in ^92^PLLLHKFLLA affected not only the formation of Bm65 aggregates but also the intracellular distribution of Bm65 in BmN cells.

### 3.3. Formation of Bm65 Aggregates Blocked by Mutations in ^92^PLLLHKFLLA

**Figure 3:**
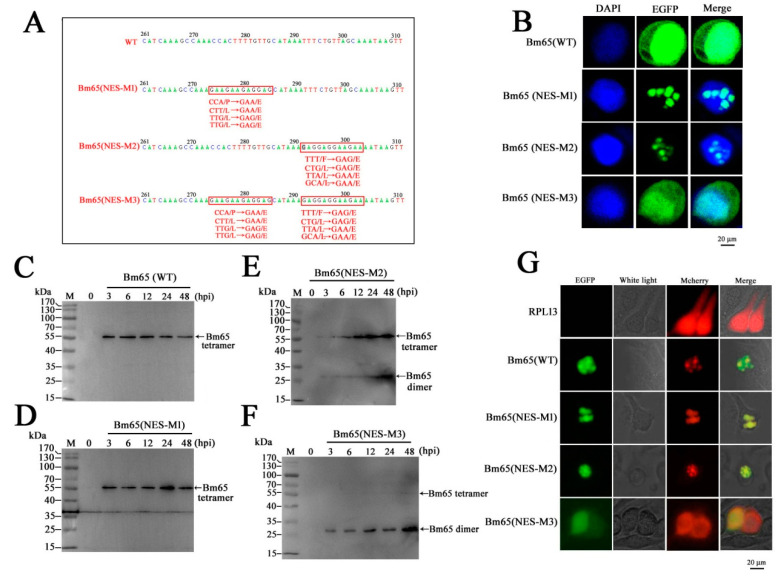
Effect of mutations in ^92^PLLLHKFLLA on the formation of the Bm65 tetramer and Bm65 aggregate. (**A**) Comparison of Bm65 sequences between wild-type and Bm65 mutant with the corresponding indications below. Amino acids were changed into glutamic acid (**E**) by point mutation as indicated by arrows. (**B**) Confocal microscopy analysis of Bm65 and Bm65 mutants in BmN cells. (**C**) Western blot analysis of wild-type Bm65 from the extracts of BmN cells infected with BmNPV. (**D**) Western blot analysis of Bm65(NES-M1). (**E**) Western blot analysis of Bm65 (NES-M2). (**F**) Western blot analysis of Bm65(NES-M3). (**G**) Confocal microscopy analysis of Bm65 and Bm65 mutant fusion with EGFP expressed in BmN cells.

To further study the role of the ^92^PLLLHKFLLA in the formation of Bm65 aggregates, three mutations, including ^92^P(E)L(E)L(E)L(E)HKFLLA, ^92^PLLLHKF(E)L(E)L(E)A(E), and ^92^P(E)L(E)L(E)L(E)HKF(E)L(E)L(E)A(E), were made in the ^92^PLLLHKFLLA region according to the instructions of the MutanBEST Kit. The Bm65 mutants were subsequently named Bm65(NES-M1), Bm65(NES-M2), and Bm65(NES-M3), respectively. In the present study, a potential NES motif in Bm65 with striking similarity to leucine-rich NES motifs was identified as a functional NES that could regulate the dynamic transport of Bm65 between the nucleus and cytoplasm. To further study the role of ^92^PLLLHKFLLA, three recombinant viruses were constructed to perform a subcellular localization analysis of Bm65 and Bm65 mutants in BmN cells. The sequencing results are shown in Figure 3. These results indicated that three mutations were successfully identified in the ^92^PLLLHKFLLA region of Bm65. Confocal fluorescence microscopy showed that EGFP was localized mainly in the nucleus in wild-type Bm65, Bm65(NES-M1), and Bm65(NES-M2) cells, but it showed a uniform distribution of fluorescence signal in BmN cells with expression of Bm65(NES-M3) (Figure 3B).

To further study the effect of mutations in the hydrophobic region on the Bm65 tetramer, recombinant Bm65-mutant viruses were constructed. Western blotting was performed to examine the expression of Bm65 mutants in BmN cells infected with recombinant viruses. The results indicated that Bm65(NES-M1) and Bm65(NES-M2) did not affect the formation of the Bm65 tetramer (Figure 3D,E), whereas the Bm65 tetramer was not detected in BmN cells infected with Bm65(NES-M3) virus (Figure 3F). As expected for Bm65(NES-M1) and Bm65(NES-M2) (Figure 3B), partial mutations in the hydrophobic region of Bm65 NES could restrain nucleocytoplasmic transport, resulting in the accumulation of Bm65 in the nucleus of BmN cells. However, it was unclear whether a uniform distribution of fluorescence signals was observed in BmN cells expressing Bm65(NES-M3). Thus, further research is required to study the mechanism of the dynamic transport of Bm65 between the nucleus and cytoplasm.

RPL13 has been reported to interact with Bm65 in BmN cells [21]. To demonstrate the effect of ^92^PLLLHKFLLA on the interaction between Bm65 and RPL13 and the formation of Bm65 aggregates, Bm65 mutant fusions with EGFP and RPL13 fusions with mCherry were co-expressed in BmN cells through co-transfection. Confocal microscopy showed that RPL13 was evenly distributed in BmN cells, but wild-type Bm65 and RPL13 co-localized in the nuclei of BmN cells in the form of aggregates, as well as Bm65(NES-M1/M2) and RPL13 (Figure 3G). The successful mutation of ^92^PLLLHKFLLA into ^92^EEEEHKEEEE abolished the interaction between Bm65 and RPL13, and the visible aggregate accumulated in the nucleus of BmN cells was also abrogated by Bm65(NES-M3). However, partial mutations in the ^92^PLLLHKFLLA region of Bm65, including Bm65(NES-M1) and Bm65(NES-M2), did not abolish the Bm65 aggregate. Some small proteins (<40 kDa) can passively diffuse between the cytoplasm and nucleus, resulting in a uniform distribution of target proteins in cells [4]. Therefore, the binding sites of Bm65(NES-M3) with RPL13 were completely disrupted and could block the reciprocal interactions between Bm65 and RPL13, resulting in a uniform distribution of Bm65(NES-M3). Furthermore, the mutation inhibited Bm65 tetramer production in BmN cells. Thus, the fluorescence signal was uniform in BmN cells through passive diffusion and in the EGFP control.

### 3.4. Inhibition of Viral Propagation by Mutations in the ^92^PLLLHKFLLA of Bm65

**Figure 4:**
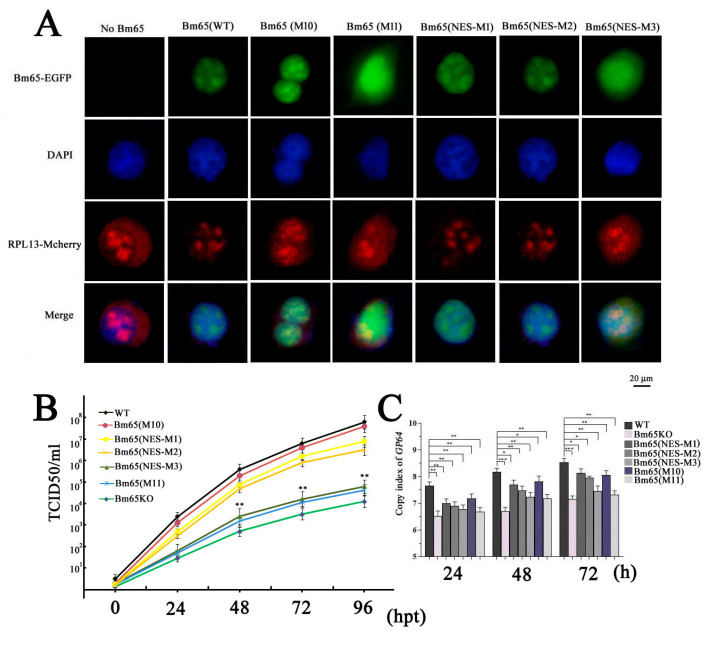
Mutations in the ^92^PLLLHKFLLA region of Bm65 sharply decreased viral propagation. (**A**) Interactions between Bm65 or Bm65 mutants and RPL13 in BmN cells via fluorescence co-localization microscopy analysis. (**B**) Virus growth curves of different viruses with mutations in Bm65 were created. (**C**) qPCR analysis was performed to determine viral genome copies to compare the replication efficiency of recombinant viruses. WT, wild-type BmNPV; Bm65KO, Bm65-deleted BmNPV; Bm65(NES-M1/M2/M3), recombinant BmNPV viruses with specific mutations in the ^92^PLLLHKFLLA of Bm65; Bm65(M10), recombinant BmNPV with mutations of ^33^R(A)R(A)I(A)K(A); Bm65(M11), recombinant BmNPV with mutations of ^33^R(A)R(A)I(A)K(A) and ^92^P(E)L(E)L(E)L(E)HKF(E)L(E)L(E)A(E). * *p* < 0.05, ** *p* < 0.01, and *** *p* < 0.001.

To demonstrate the correlation between Bm65 aggregates and the interaction between Bm65 and RPL13, co-transfection of BmN cells with two recombinant plasmids was performed to express RPL13 and Bm65 or Bm65 mutants, respectively. RPL13 and Bm65 were fused with mCherry and EGFP, respectively. Thus, the intracellular distribution of green and red fluorescence using confocal microscopy was used to examine the co-localization of Bm65 and RPL13 in BmN cells. The results showed fluorescence co-localization between RPL13 and wild-type Bm65 or the three Bm65 mutants, including Bm65(M10), Bm65(NES-M1), and Bm65(NES-M2) (Figure 4A). However, Bm65(M11) and Bm65(NES-M3) of Bm65 mutants did not co-localize with RPL13 in BmN cells (Figure 4A). The mutation of ^92^PLLLHKFLLA into ^92^EEEEHKEEEE completely blocked the interaction between RPL13 and Bm65(M11) or Bm65(NES-M3), resulting in the failure of fluorescence co-localization and Bm65 aggregation in BmN cells.

The TCID50 endpoint dilution in BmN cells was measured to determine the titers of the recombinant viruses. The results indicated that recombinant viruses with the mutations of ^92^EEEEHKEEEE in the Bm65 sequence all showed a decreased tendency in the slope of the growth curves compared to the WT, Bm65(M10), Bm65(NES-M1), and Bm65(NES-M2) viruses (Figure 4B). The statistical analysis revealed a significant difference in the viral titers between Bm65 (NES-M3) or Bm65(M11) and the Bm65 wild-type virus from 48 to 96 hpt. To further study the effect of mutations in the ^92^PLLLHKFLLA region of Bm65 on viral propagation, several recombinant viruses were used to infect BmN cells to compare the viral genome copies. Mutations in the ^92^PLLLHKFLLA gene were introduced into the genomes of the Bm65(M11), Bm65(NES-M3), and Bm65KO recombinant viruses. For this purpose, qPCR analysis was performed to examine the copies of *gp64* to compare the efficiency of viral propagation, and the copies of *gp64* were also used to analyze the effect of the mutations on viral propagation. Compared to the Bm65, Bm65(M10), Bm65(NES-M1), and Bm65(NES-M2), the mutations in the ^92^PLLLHKFLLA of Bm65(M11), Bm65(NES-M3), and Bm65KO viruses obviously inhibited viral propagation, resulting in a decrease in the *gp64* copy number (Figure 4C). There was a significant difference (*p* < 0.001) in *gp64* copy numbers between Bm65 and Bm65(M11), as well as between Bm65 and Bm65(NES-M3).

## 4. Discussion

BmNPV, a typical baculovirus with a circular double-stranded DNA genome, can quickly spread viral infection among silkworms on a large scale, causing great economic loss. Silkworms have evolved some effective strategies for combating an invading microorganism in long-term evolutionary processes [23,24], and some silkworm proteins have been reported to participate in the resistant response to viral invasion and propagation [25]. BmNPV replication and propagation partly depend on the complicated interaction between the virus and silkworm. Bm65 is a BmNPV-encoded protein with a small weight of 12.2 kDa. In theory, Bm65 should exhibit a uniform distribution in BmN cells according to cell size. However, Bm65 exists mainly in the nuclei of BmNPV-infected BmN cells, and it can spontaneously form Bm65 aggregates accumulated in the nucleus in the late stages of viral infection. In addition, hydrophobic amino acids have been shown to promote the accumulation of Bm65 aggregates in the nucleus. We aimed to reveal the role of ^33^RRIK in the nuclear entry of Bm65 via point mutation. The subcellular localization results showed that the mutations in ^33^RRIK did not block the nuclear entry of the Bm65 mutant, but clearly accelerated the accumulation of Bm65 in the nucleus of BmN cells (Figure 2B). However, the components of Bm65 aggregates remain unclear. Therefore, it is essential to specify the specific components of Bm65 aggregates and define their roles in viral propagation. Meanwhile, it will be helpful for us to further demonstrate the interactions between Bm65 and host proteins, as well as the mechanism of Bm65 aggregate formation.

Some ribosomal proteins are not only the requisite components of ribosomes but also directly participate in the regulation of host immunity and viral propagation. To date, diverse ribosomal proteins have been reported to interact with viral proteins to regulate viral biosynthesis, viral replication, and host immune responses. For example, RPL4 interacts with VP3 to regulate the replication of infectious bursal disease virus (IBDV) [26]. RPL18 is a well-known critical factor due to its interaction with many viral proteins from Arabidopsis thaliana, Ebola viruses, and dengue viruses [6]. RPL13 promotes IRES-driven translation of foot and mouth disease in a DDX3-dependent helicase [27]. Guan et al. reported that RPL13 participates in the innate immune response induced by foot-and-mouth disease virus [28]. Therefore, ribosomal proteins are a class of multifunctional proteins that play diverse roles during viral replication, virion assembly, and the antiviral immune response. In general, the viral strategy exploits host cellular resources for the production and spread of progeny virions. Some host cell proteins directly participate in viral replication, assembly, and maturation processes. In addition, some host proteins can be hijacked to inhibit antiviral immune responses, thereby facilitating viral propagation. We considered that BmNPV hijacking RPL13 could facilitate viral replication to improve viral production, which was confirmed by qPCR and TCID50 analyses (Figure 4).

Zhang et al. previously identified RPL13 as a stress-inducible gene that plays a regulatory role in NF-κB signaling [29]. Moreover, RPL13 participates in viral translation and host innate immune response [27,28]. Recently, RPL13 was reported to interact with Bm65 to repair UV-induced DNA damage [21]. Therefore, RPL13 could function as an important binding factor with viral protein to regulate the interactions between BmNPV and silkworms. This also demonstrates the scientific correlation between the interactions and Bm65 aggregates.

In the present study, the hydrophobic region of ^92^PLLLHKFLLA was identified as an efficient NES of Bm65 and was involved in the dynamic transport of Bm65 from the nucleus to the cytoplasm (Figure 1). Furthermore, a complete mutation in the ^92^PLLLHKFLLA region could block the interaction between Bm65 and RPL13, resulting in the failure of Bm65 aggregate production in the nucleus. Additionally, Western blot analysis confirmed that the mutations inhibited the formation of the Bm65 tetramer and viral propagation. ^76^KRKCSK is an efficient nuclear localization signal for nuclear import of Bm65, but Bm65 mutants that lose the ability to form stable protein complexes (>40 kDa) could not stably exist in the nuclei of BmN cells. Therefore, it is reasonable to speculate that the uniform distribution of the Bm65 mutant in BmN cells occurs through passive diffusion. However, the specific components of Bm65 aggregates and their roles in viral propagation remain unclear, and further research is required to clarify these issues.

## Figures and Tables

**Table 1:** Primers, plasmids, and viruses used in the study.

Primers	Primer Sequence (5′-3′)	Enzyme Digestion Site	Plasmids	Viruses
Bm65NES-F	ATGAATTCATGTTGCGTCTCATCAAAGCCA	EcoRI	HTB-P_ie1_-Bm65NES-EGFP	Wild-type BmNPV
Bm65NES-R	ATCTCGAGTTACTTGTACAGCTCGTCCATGC	XhoI	HTB-P_ie1_-Bm65NES(M3)-EGFP	vBm^Bm65(NES-M1)^
Bm65NES(M3)-R	TACTGCAGCAACTTATTTTCTTCCTCCTCTTTA	PstI	HTB-P_ie1_-EGFP	vBm^Bm65(NES-M2)^
Bm65-flag-F	CGACTAGTCGGCCAACATATTCAATTACATGGCCGAGCT	Spe I	HTB-P_ie1_-Bm65-EGFP	vBm^Bm65(NES-M3)^
NES-M1-R	TACTCGAGTTACTTATCGTCGTCATCCTTGTAATCCAACTTATTTGCTAACA	Xho I	HTB-P_ie1_-RPL13-HA	vBm^Bm65M10^
NES-M2-R	TACTCGAGTTACTTATCGTCGTCATCCTTGTAATCCAACTTATTTTCTTCCT	Xho I	HTB-P_ie1_-Bm65-Flag	vBm^Bm65M11^
Bm65-flag-F	ATGAATTCATGGCGACGACTCTGTACACCAACA	Xho I	pMD18T-P_Bm65_-Bm65-flag	vBm^Bm65KO-GFP^
Bm65-flag-R	ATCTCGAGTTACTTATCGTCGTCATCCTTGTAATCCAACTTATTTGCTAACA	EcoRI	HTB-P_ie1_-egfp-sv40-P_Bm65_-Bm65-flag	
M13-F	GTTTTCCCAGTCACGAC	Xho I	HTB-P_ie1_-Bm65-EGFP	
M13-R	CAGGAAACAGCTATGAC	EcoRI	pMD18T-Bm65	
Bm65(M1)-F	ACCTTAACGCACGCATAAAACAGCA	Xho I	pMD18T-33R(A)/Bm65	
Bm65(M2)-F	ACCTTAACAGAGCCATAAAACAGCATTCGA	---	pMD18T-34R(A)/Bm65	
Bm65(M3)-F	ACCTTAACAGACGCGCAAAACAGCA	---	pMD18T-35I(A)/Bm65	
Bm65(M4)-F	ACCTTAACAGACGCATAGCACAGCATTC		pMD18T-36K(A)/Bm65	
Bm65(M5)-F	ACCTTAACGCAGCCATAAAACAGCA		pMD18T-33R(A)34R(A)/Bm65	
Bm65(M6)-F	ACCTTAACAGAGCCGCAAAACAGCA		pMD18T-34R(A)35I(A)/Bm65	
Bm65(M7)-F	ACCTTAACAGACGCGCAGCACAGCA		pMD18T-35I(A) 36K(A)/Bm65	
Bm65(M8)-F	ACCTTAACGCAGCCGCAAAACAG		pMD18T-33R(A) 34R(A) 35I(A)/Bm65	
Bm65(M9)-F	ACCTTAACAGAGCCGCAGCACAGCA		pMD18T-34R(A) 35I(A)36K(A)/Bm65	
Bm65(M10)-F	ACCTTAACGCAGCCGCAGCACAGCA		pMD18T-33R(A)34R(A)35I(A) 36K(A)/Bm65	
Bm65M-R	TGCTCGTGATGCCCGTGTACAATTT		HTB-P_ie1_-egfp-sv40-P_Bm65_-Bm65(M10) -flag	
Bm65-F	ATGAATTCATGGCGACGACTCTGTACACCA	EcoRI	HTB-P_ie1_-egfp-sv40-P_Bm65_-Bm65(M11) -flag	
Bm65-R	ATCTGCAGCAACTTATTTGCTAACAGAAATTTATGCA	Pst I		
Bm65(M11)-F	GCAGCACAGCATTCGAACAAACAAG			
Bm65(M11)-R	GGCTGCGTTAAGGTTGCTCGTGAT			
qPCR-F	CGATGCGGCGTTTCTAC			
qPCR-R	GTTGCCCTCAGCGTCCA			

Note: underlined letters indicate restriction enzyme digestion sites.

## Data Availability

All required data are available in the manuscript. Additional data can be provided upon request.
